# Circulating Annexin A5 Levels after Atrial Switch for Transposition of the Great Arteries: Relationship with Ventricular Deformation and Geometry

**DOI:** 10.1371/journal.pone.0052125

**Published:** 2012-12-20

**Authors:** Clare T. M. Lai, Pak-cheong Chow, Sophia J. Wong, Koon-wing Chan, Yiu-fai Cheung

**Affiliations:** Department of Paediatrics and Adolescent Medicine, The University of Hong Kong, Hong Kong, China; Instituto Butantan, Brazil

## Abstract

**Background:**

Inflammatory cytokines, cardiomyocyte apoptosis, and altered collagen turnover may contribute to unfavourable ventricular remodeling. This unfavourable ventricular remodelling is well documented in patients after atrial switch operation for complete transposition of the great arteries. We therefore tested if levels of circulating markers of inflammation, apoptosis, collagen synthesis, and extracellular matrix degradation are altered in patients after atrial switch operation for transposition of the great arteries.

**Methods and Results:**

Circulating tumour necrosis factor (TNF)-α, annexin A5 (AnxA5), carboxy-terminal propeptide of type I procollagen (PICP), amino-terminal propeptide of type III procollagen (PIIINP), matrix metalloproteinase-1 (MMP-1), and tissue inhibitor of metalloproteinase-1 (TIMP-1) levels were determined in 27 patients aged 25.2±3.1 years and 20 controls. Ventricular myocardial deformation and left ventricular eccentricity index (EI) were determined by speckle tracking and two-dimensional echocardiography, respectively. Compared with controls, patients had significantly higher circulating AnxA5 (p<0.001) and TNF-α (p = 0.018) levels, but similar PICP, PIIINP, MMP-1 and TIMP-1 levels. For the whole cohort, plasma AnxA5 correlated with serum TNF-α (p = 0.002), systemic ventricular global longitudinal strain (GLS) and systolic and early diastolic strain rate (all p<0.001), and subpulmonary ventricular GLS and early diastolic strain rate (both p<0.001). In patients, plasma AnxA5 level correlated positively with subpulmonary ventricular EI (p = 0.027). Multiple linear regression analysis identified systemic ventricular GLS (β = −0.50, p<0.001) and serum TNF-α (β = 0.29, p = 0.022) as significant correlates of plasma AnxA5.

**Conclusions:**

Elevated plasma AnxA5 level in patients after atrial switch operation is associated with impaired systemic myocardial deformation, increased subpulmonary ventricular eccentricity, and increased serum TNF-α level.

## Introduction

Progressive systemic right ventricular (RV) dysfunction in patients after atrial switch operation for complete transposition of the great arteries (TGA) is well documented [1]–[4]. Dilation of the systemic right ventricle, alteration of septal geometry, and compression of the subpulmonary left ventricle provide the basis for adverse ventricular-ventricular interaction [5], [6]. Proposed mechanisms of systemic RV dysfunction after Senning or Mustard procedure include impaired myocardial perfusion [7], myocardial fibrosis [8], and onset of tricuspid regurgitation [1]–[4].

The role of inflammatory cytokines in the development of heart failure in congenital heart disease is increasingly unveiled. In adults with congenital heart disease, elevation of serum tumour necrosis factor (TNF)-α level has been associated with severity of heart failure symptoms [9]. Importantly, TNF-α has been shown in vitro to provoke apoptosis of cardiomyocytes [9]–[11], decrease collagen synthesis in cardiac fibroblasts [12], and activate matrix metalloproteinases to degrade extracellular matrix [13], [14], all of which may contribute to unfavourable ventricular remodeling. Experimental data suggest involvement of annexin A5 (AnxA5) in cardiomyocyte apoptosis. [15] In hypertensive subjects, plasma AnxA5 level is increased and shown to correlate inversely with left ventricular (LV) systolic function [16]. Importantly, plasma and myocardial AnxA5 levels correlate strongly and increase in a dose-dependent manner with worsening hypertension and histological evidence of cardiomyocyte apoptosis [16]. In patients with heart failure undergoing cardiac resynchronization therapy, a higher baseline plasma AnxA5 level is associated with worse LV ejection fraction [17]. In adults with heart failure related to hypertension and dilated cardiomyopathy, circulating biomarkers of collagen synthesis [18], [19] and extracellular matrix degradation [20]–[22] have been reported. Whether these biomarkers are altered in patients who are at risk of systemic RV dysfunction after atrial switch operation has hitherto not been explored.

In the present study, we determined the circulating levels of TNF-α, AnxA5, and biomarkers of collagen synthesis and extracellular matrix degradation including carboxy-terminal propeptide of type I procollagen (PICP), amino-terminal propeptide of type III procollagen (PIIINP), matrix metalloproteinase-1 (MMP-1), and tissue inhibitor of metalloproteinase-1 (TIMP-1) in patients after atrial switch operation for complete TGA. We further explored the relationships of these circulating biomarkers with ventricular myocardial deformation and geometry.

## Materials and Methods

### Ethics Statement

All subjects gave written informed consent to participate in this study approved by the Institutional Review Board of The University of Hong Kong/Hospital Authority West Cluster, Hong Kong.

### Subjects

Twenty-seven patients with complete TGA who had undergone Mustard or Senning procedure were recruited consecutively from the cardiac clinic. Of these 27 patients, 18 patients had been evaluated previously in a study on diastolic ventricular interaction conducted 2 years ago [6] and they were invited again to have another echocardiographic evaluation and blood examination as detailed below. The following data were obtained from the case notes: demographic variables, age at and type of operation, duration of follow-up since surgery, and current cardiac medications. Twenty-two had undergone Senning operation while 5 had Mustard procedure. Nine patients had closure of an associated ventricular septal defect, 2 of whom had a small residual defect. Mild baffle leak was found in 1 patient. Tricuspid regurgitation was absent in 7 patients, trace to mild in 19, and severe in 1. Mild subpulmonary stenosis with a Doppler-derived systolic gradient of about 30 mmHg was noted in 2 patients. Cardiac arrhythmias were documented in 5 patients, 2 of whom had sinus node dysfunction, and 1 each of first degree heart block, atrial flutter, and infrequent ventricular ectopics. All of the patients were having a sinus rhythm at the time of echocardiographic assessment. At the time of study, 4 patients were taking cardiac medications, which included angiotensin-converting enzyme inhibitor or angiotensin receptor antagonist in 4 patients, beta-blocker in 3, digoxin in 2, and furosemide, spironolactone, sotalol, and aspirin in 1. Twenty healthy subjects were recruited as controls. These included healthy adult volunteers and subjects followed up in cardiac clinic for non-specific chest pain and palpitation but without documented organic causes. The body weight and height of all subjects were measured, and the body mass index and body surface area were calculated accordingly.

### Echocardiographic Assessment

Transthoracic echocardiography was performed using the Vivid 7 ultrasound system (General Electric Vingmed Ultrasound, Horten, Norway). The echocardiographic data were recorded in digital versatile discs and analyzed offline using EchoPAC software (General Electric, Horten, Norway). Measurements of echocardiographic parameters were made over three cardiac cycles and the average was used for statistical analysis.

From the apical four-chamber view, two-dimensional speckle tracking echocardiography was performed to determine global longitudinal myocardial deformation of the systemic and subpulmonary ventricles as described previously [6], [23]. The entire contour of the respective ventricle was traced for determination of systolic global longitudinal strain (GLS) and global longitudinal strain rate during systole (SRs), early diastole (SRe), and late diastole (SRa). Our group has previously reported on a low intra- and inter-observer variability for measurement of GLS [23].

From the parasternal short-axis view, the subpulmonary left ventricular (LV) geometry of patients was assessed by calculating the diastolic eccentricity index, defined as D2/D1 where D1 is the maximum distance from the surface of the mid-ventricular septum to that of the LV free wall and D2 is the antero-posterior distance measured perpendicular to D1 [24].

### Assay of Biomarkers

Venous blood was obtained after echocardiographic assessment and centrifuged immediately after collection. The serum and plasma samples were stored at −80°C until assay. Patient and control samples were run in the same batches to minimize inter-batch variations. Serum TNF-α level was determined by enzyme-linked immunoassay (Quantikine HS; R&D System, Minneapolis, USA). The standard range is 0.5–32 ng/ml and the sensitivity is 0.11 ng/ml. Plasma Annexin A5 was measured using the commercially available Annexin V-specific ELISA (Zymutest Annexin V, Hyphen BioMed, France) with a standard range of 0.5–10.7 µg/l and a sensitivity of 0.1 µg/l. The enzymatic activities of serum MMP1 and TIMP1 were measured using the Biotrak ELISA system (Amersham, GE Healthcare, Buckinghamshire, UK). For MMP1 assay, the standard range is 6.25–100 ng/ml with a sensitivity of 1.7 ng/ml, while for TIMP1, the standard range is 3.13–50 ng/ml with a sensitivity of 1.25 ng/ml. Serum PICP was measured with immunoassay (Takara Biochemicals Co, Osaka, Japan) with a standard range of 10–640 µg/l and a sensitivity of 2 µg/l. The plasma PIIINP was measured using radioimmunoassay (Orion Diagnostica, Espoo, Finland) with a standard range of 1–50 µg/l and a sensitivity of 0.3 µg/l. All measurements were performed in duplicate and the mean was used for further analysis.

### Statistical Analysis

All data are presented as mean±standard deviation. The demographic variables, echocardiographic parameters, and circulating biomarkers of patients and controls were compared using unpaired Student’s t test. Systemic RV strain indices in patients were compared with systemic LV indices in controls, while subpulmonary LV indices in patients were compared with subpulmonary RV indices in controls. Relationships of the circulating biomarkers found to differ significantly between patients and controls with ventricular strain indices and geometry were assessed using Pearson correlation analysis. Multiple linear regression analysis was further used to identify independent determinants of circulating biomarkers found to be significantly elevated in patients and related to indices of ventricular myocardial deformation. Statistical analyses were performed with SPSS, version 16.0 (SPSS Inc., Chicago, IL, USA). A p value less than 0.05 was considered statistically significant while Bonferroni adjustment was made for multiple correlational analyses.

## Results

### Worse Ventricular Deformation and Greater LV Eccentricity in Patients

In order to confirm that patients after atrial repair for TGA have unfavourable ventricular mechanics and remodelling, 27 patients (17 males) aged 25.2±3.1 years at 23.7±2.6 years after atrial switch operation were recruited for evaluation of ventricular myocardial deformation and LV eccentricity. The findings were compared with those of 20 control subjects (11 males) aged 25.5±3.8 years (p = 0.75). The body mass index (21.8±3.2 kg/m^2^ vs 21.8±3.3 kg/m^2^, p = 0.96) and body surface area (1.7±0.2 m^2^ vs 1.7±0.1 m^2^, p = 0.39) were similar between patients and controls. By echocardiographic assessment, patients had significantly reduced systemic ventricular GLS and global systolic and diastolic strain rates (all p<0.001) compared with controls (Table 1). For the subpulmonary ventricle, the ventricular GLS (p<0.001) and SRe (p = 0.001) were also significantly lower in patients than controls. The morphologic left ventricle was significantly more eccentric in patients than controls (1.9±0.6 vs 1.0±0.1, p<0.001).

**Table 1:** Indices of global myocardial deformation of the systemic and subpulmonary ventricles in patients and controls.

Ventricular myocardial deformation indices	Patients(n = 27)	Controls(n = 20)	p
*Systemic ventricle*			
GLS (%)	11.3±2.2	17.4±3.7	<0.001*
SRs (/s)	0.60±0.11	0.97±0.20	<0.001*
SRe (/s)	0.72±0.18	1.37±0.46	<0.001*
SRa (/s)	0.30±0.10	0.54±0.24	<0.001*
*Subpulmonary ventricle*			
GLS (%)	15.1±3.7	21.2±5.8	<0.001*
SRs (/s)	1.03±0.28	1.11±0.25	0.27
SRe (/s)	1.05±0.28	1.46±0.43	0.001*
SRa (/s)	0.49±0.14	0.73±0.72	0.16

GLS indicates global systolic longitudinal stain; SRa, global late diastolic strain rate; SRe, global early diastolic strain rate; SRs, global systolic strain rate.

* statistically significant.

### Plasma AnxA5 and Serum TNF-α Levels were Higher in Patients than Controls

**Figure 1:**
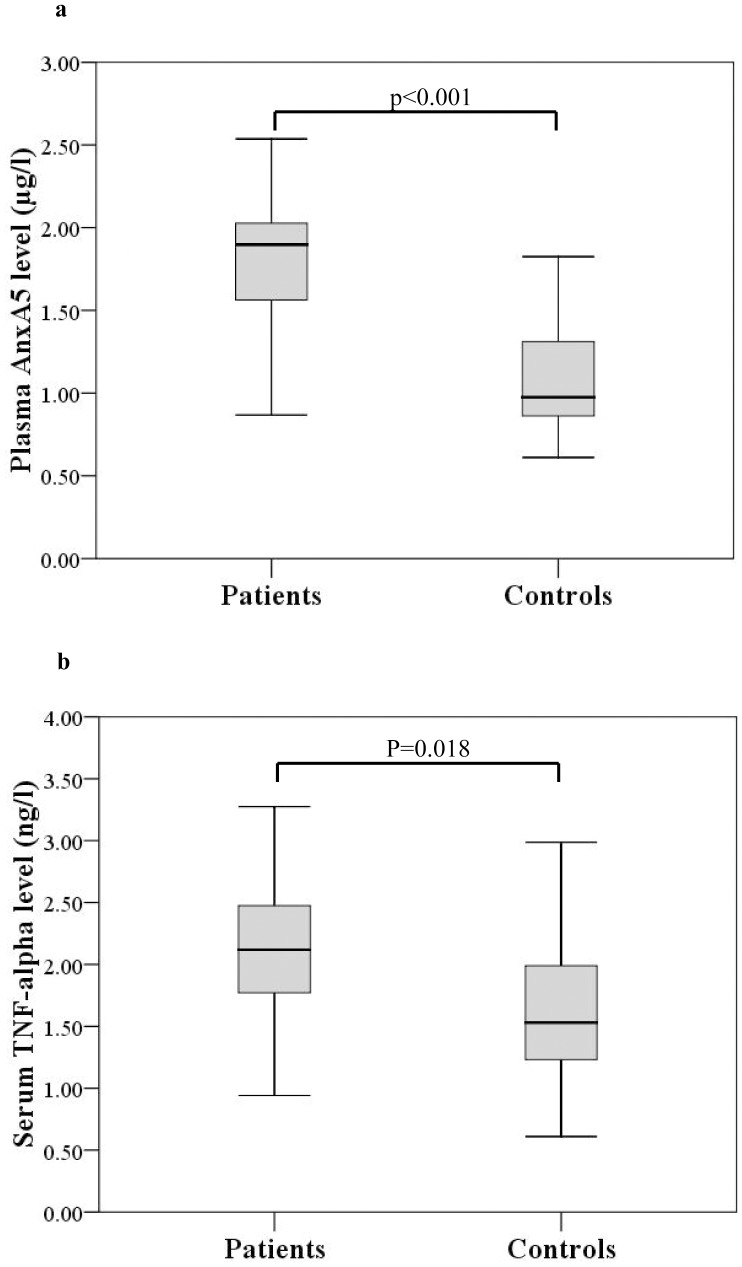
Box-plots showing (a) plasma annexin A5 (AnxA5) levels and (b) serum tumour necrosis-factor (TNF)-α levels in patients and controls.

Patients had significantly higher plasma AnxA5 (p<0.001) and serum TNF-α (p = 0.018) levels than controls (Fig. 1). On the other hand, the circulating levels of PICP (253.4±71.4 ng/ml vs 275.6±74.9 ng/ml, p = 0.31), PIIINP (3.9±1.5 ng/ml vs 4.4±1.2 ng/ml, p = 0.25) MMP-1 (39.5±9.9 ng/ml vs 34.5±13.6 ng/ml, p = 0.16), and TIMP-1 (153.7±36.5 ng/ml vs 156.7±70.1 ng/ml, p = 0.85) were similar between patients and controls.

Within the patient group, there were no significant differences in the levels of these biomarkers between patients with and those without additional VSD repair. However, patients on cardiac medications had significantly higher plasma AnxA5 level (2.4±0.6 µg/ml vs 1.8±0.5 µg/ml, p = 0.034) and lower subpulmonary ventricular GLS (13.3±0.7% vs 15.5±3.9%, p = 0.025) and tended to have lower systemic RV GLS (9.7±1.3% vs 11.6±2.2%, p = 0.13) compared with those not on medical treatments.

### Significant Correlates of Plasma AnxA5 Level

**Figure 2:**
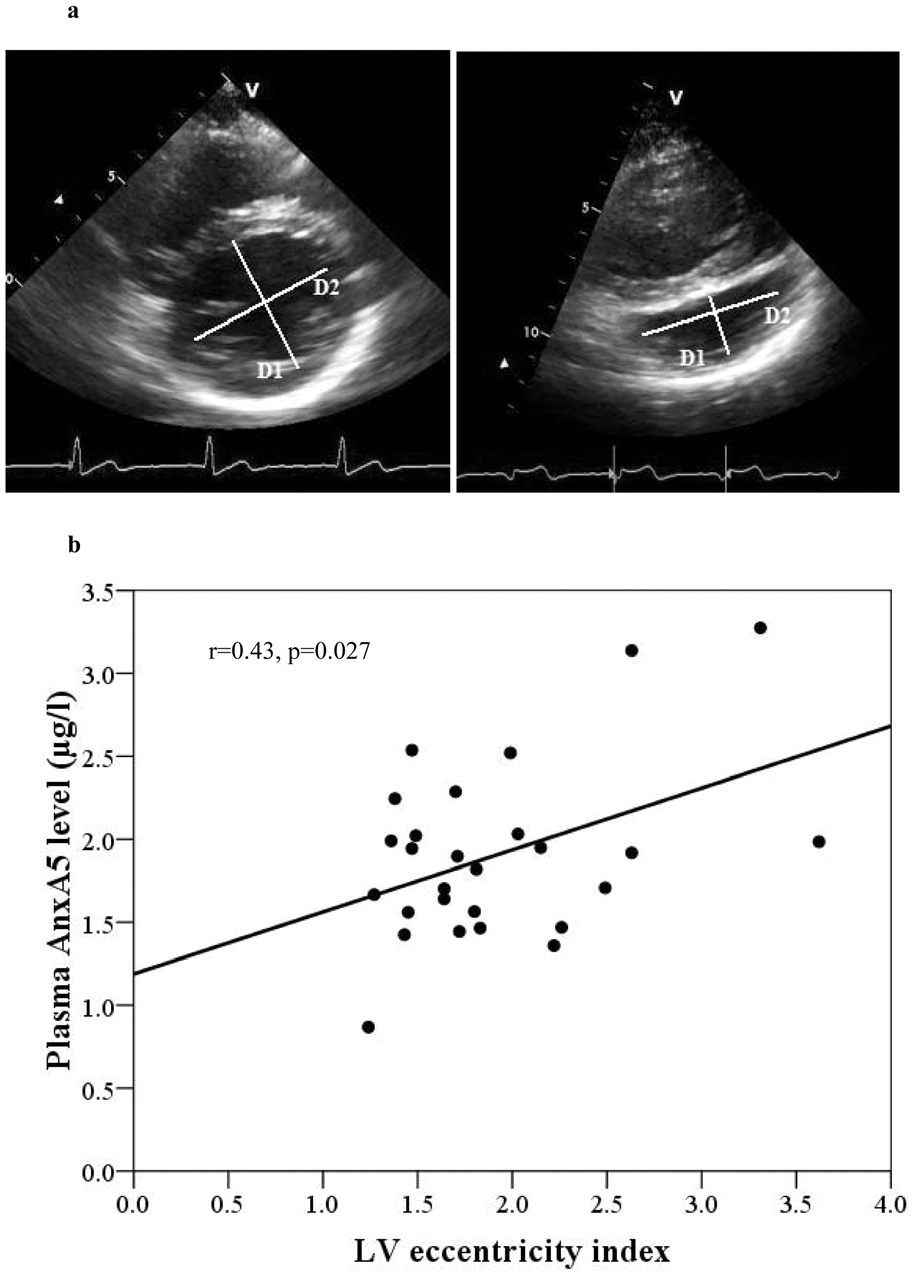
(a) Derivation of eccentricity index from a patient (right) and a control subject (left). D1 is the maximum distance from the surface of the mid-ventricular septum to that of the LV free wall and D2 is the antero-posterior distance measured perpendicular to D1. (b) Scatter plot showing significant correlation between subpulmonary left ventricular (LV) eccentricity index and plasma annexin A5 (AnxA5) level.

For the whole cohort, plasma AnxA5 level correlated negatively with systemic ventricular GLS (p<0.001), SRs (p<0.001), and SRe (p<0.001), and subpulmonary ventricular GLS (p<0.001) and SRe (p<0.001) (table 2). Among patients, plasma AnxA5 correlated significantly with subpulmonary LV eccentricity index (r = 0.43, p = 0.027) (Fig. 2), with the correlation remaining the same even with exclusion of the one patient with severe tricuspid regurgitation.

**Table 2:** Correlations between plasma annexin A5 (AnxA5) and serum tumour necrosis factor (TNF)-α and parameters of ventricular myocardial deformation.

	AnxA5		TNF-α	
	r	p	r	p
*Systemic ventricle*				
GLS	−0.58	<0.001*	−0.25	0.095
SRs	−0.51	<0.001*	−0.27	0.071
SRe	−0.53	<0.001*	−0.26	0.081
SRa	−0.31	0.033	−0.21	0.17
*Subpulmonary ventricle*				
GLS	−0.55	<0.001*	−0.27	0.068
SRs	−0.28	0.053	−0.17	0.25
SRe	−0.52	<0.001*	−0.36	0.013
SRa	−0.26	0.077	−0.18	0.24

Abbreviations as in Table 1.

* statistically significant after Bonferroni correction.

**Figure 3:**
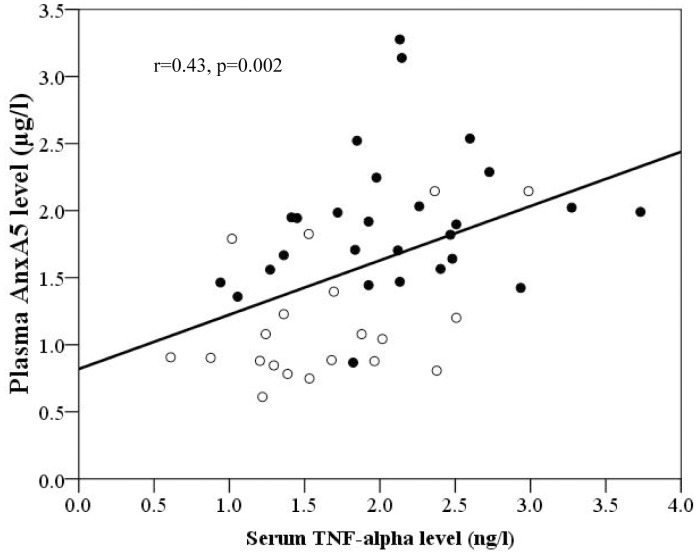
Scatter plot showing significant positive correlation between serum tumour necrosis factor (TNF)-α level and plasma annexin A5 (AnxA5) levels (solid circles represent patients and empty circles represent controls).

Serum TNF-α level correlated positively with plasma AnxA5 level for the whole cohort (r = 0.43, p = 0.002) (Fig. 3). However, there were no correlations between serum TNF-α level and parameters of systemic or subpulmonary indices of ventricular myocardial deformation (Table 2).

Multiple stepwise linear regression of the entire cohort was performed to identify significant correlates of plasma AnxA5 level. The independent covariates entered into the model were age, sex, body mass index, systemic and subpulmonary ventricular GLS, SRs, SRe, and SRa, and serum TNF-α level. The significant independent correlates were systemic ventricular GLS (β = −0.50, p<0.001) and serum TNF-α level (β = 0.29, p = 0.022). When only the patient cohort was analyzed with addition of duration after operation, subpulmonary LV eccentricity and the need for cardiac medications to the same list of covariates, the significant determinants of plasma AnxA5 identified were LV eccentricity index (β = 0.39, p = 0.033) and use of cardiac medications (β = 0.37, p = 0.041).

## Discussion

In the present study, we have demonstrated 1) significant elevation of plasma AnxA5 level in Senning and Mustard patients compared with health subjects, 2) a positive correlation between plasma AnxA5 level and serum TNF-α concentration, and 3) systemic ventricular strain, subpulmonary LV eccentricity, and the use of cardiac medications to be significant determinants of plasma AnxA5 level.

Elevation of serum TNF-α in our patients is consistent with findings of previous studies [9], [25]. In patients with a variety of congenital heart lesions including functional single ventricles, tetralogy of Fallot, and systemic right ventricles, Sharma et al documented elevation of TNF-α level and its association with worse functional status [9]. In patients with aortic stenosis, serum TNF-α level has similarly been shown to be increased compared with healthy subjects and related to functional class. [25] Provocation of cardiomyocyte apoptosis and cardiac remodeling have been demonstrated in vitro through activation of multiple cell death pathways by TNF-α [10]. This may be of relevance in patients after atrial switch operation who are at risk of systemic RV dysfunction [1]–[4]. Our further demonstration of a positive correlation between circulating levels of AnxA5 and serum TNF-α is of clinical interest.

To our knowledge, this is first study to determine plasma AnxA5 level and its relationship with ventricular function and geometry in patients with a systemic right ventricle after atrial switch operation for complete TGA. Clinical studies on the levels and implications of plasma AnxA5 in cardiac patients are limited. Increased plasma AnxA5 has been reported in patients with myocardial infarction [26]–[28] and unstable angina [28]. Circulating AnxA5 can be released from injured myocardial tissue, vascular endothelial and smooth muscle cells, secretor cells of the liver and spleen, or apoptotic particles derived from circulating blood cells, and the level of which has been shown to reflect severity of cell damage [16], [26]–[32]. The origin of increased circulating AnxA5 in our patients is, however, unclear. In hypertensive patients with heart failure, release of AnxA5 from the heart has been suggested by the existence of a coronary sinus-peripheral venous AnxA5 concentration gradient [16]. Whether this gradient exists in patients atrial switch operation with systemic RV dysfunction undoubtedly requires further study for clarification. Nonetheless, the finding of inverse correlations between plasma AnxA5 and systolic and diastolic ventricular strain and strain rate parameters (table 2) in our patients perhaps lend support to the heart being a potential source. The correlations between serum TNF-α levels with AnxA5 levels but not with myocardial deformation parameters warrant some comments. As alluded to earlier, TNF-α may act more upstream to provoke cardiomyocyte apoptosis and cardiac remodeling, [10] while AnxA5 may represent a more downstream marker of this remodeling process and hence reflect more strongly on the functional alteration of the systemic and subpulmonary ventricles in our patients.

The mechanism underlying increased circulating AnxA5 in our patients remains speculative. Stretch-induced up-regulation of AnxA5 has been shown in non-cardiac cells [33], [34]. The finding of a correlation between subpulmonary LV eccentricity and circulating AnxA5 level is perhaps consistent with this possibility. A greater eccentricity of the subpulmonary left ventricle in patients after atrial switch operation is a reflection of progressive systemic RV dilation [5], [6]. In a rodent model of pulmonary hypertension, progression RV dilation and dysfunction are paralleled by evidence of RV apoptosis including increased ^99m^Tc-annexin uptake and terminal deoxynucleotidyl-transferase-mediated dUTP nick-end labelling [35]. Interestingly, in heart failure patients responding to cardiac resynchronization therapy, reverse LV remodeling is associated with reduction in plasma AnxA5 [17]. It is tempting, therefore, to speculate that circulating AnxA5 may possibly originate from the stretched dysfunctional systemic right ventricle rather than the compressed left ventricle in patients after atrial switch operation. Whether RV apoptosis plays a role in progressive systemic RV dysfunction in Senning and Mustard patients is a topic for further research.

Apart from the quantity of functioning contractile units, integrity of the architectural scaffold of the myocardium is of paramount importance for normal cardiac performance. Increased levels of PICP and PIIINP are found in conditions associated with increased systemic left ventricular afterload and dilated cardiomyopathy [36], [37]. In Senning and Mustard patients, late gadolinium enhancement in cardiac magnetic resonance suggests increased myocardial fibrosis of the systemic right ventricle [8]. Hence, our finding of the absence of increased circulating biomarkers of collagen biosynthesis is surprising. It is worthwhile noting, however, that late gadolinium enhancement of myocardial segments probably represents a static state of regional fibrosis [38] while circulating PICP and PIIINP may provide a means of real-time monitoring collagen synthesis and hence the dynamics of collagen turnover [36]. The implication of reduced myocardial collagen synthesis in our young adult patient cohort at the time of study is potential decline in myocardial matrix tensile strength with the consequence of progressive systemic RV dilation and systolic dysfunction. In this regard, TNF-α has been shown to decrease collagen synthesis in cardiac fibroblasts in vitro [12] and reduced serum levels of PICP and PIIINP have been reported, albeit uncommonly, in patients with dilated cardiomyopathy [39].

Several limitations of this study warrant comments. Firstly, the origin of the various circulating biomarkers assayed in our patients remains to be identified. Secondly, potential confounding influences of cardiac medications on the levels of circulating biomarkers can not completely be excluded. Nonetheless, only 4 of the 27 patients were on cardiac medications at the time of study and we have further performed subgroup analysis based on the use of cardiac medications. Finally, it would have been ideal to determine the relationship between haemodynamic disturbances as tricuspid regurgitation and subpulmonary stenosis and levels of circulating biomarkers in a larger patient cohort.

In conclusion, this study provides the first evidence of elevated plasma AnxA5 level in patients after atrial switch operation, which is associated with impaired systemic myocardial deformation and increased serum TNF-α level. The pathophysiologcal significance of an increase in circulating AnxA5 in this adult congenital heart population, however, requires further clarification. Serial longitudinal measurement of plasma AnxA5 level with simultaneous evaluation of clinical status and ventricular function of our patients may shed more light on the potential role of this circulating marker in the monitoring of systemic RV function and prognostication.
